# Will Women Executives Reduce Corruption? Marginalization and Network Inclusion

**DOI:** 10.1177/0010414020970218

**Published:** 2020-12-02

**Authors:** Monika Bauhr, Nicholas Charron

**Affiliations:** 1 University of Gothenburg, Gothenburg, Sweden

**Keywords:** corruption and patronage, women representation, subnational politics

## Abstract

While recent studies find a strong association between the share of women in elected office and lower levels of corruption, we know less about if women in executive office cause reductions in corruption levels, and if such effects last over time. This study suggests that women mayors reduce corruption levels, but that the beneficial effect may be weakened over time. Using both regression discontinuity and first difference designs with newly collected data on French municipal elections combined with corruption risk data on close to all municipal contracts awarded between 2005 and 2016, we show that women mayors reduce corruption risks. However, newly elected women mayors drive the results, while gender differences are negligible in municipalities where women mayors are re elected. Our results can be interpreted as providing support for marginalization theories, but also suggest that the women that adapt to corrupt networks survive in office.

## Introduction

While the available evidence on the detrimental effect of corruption for human well-being and economic development has increased, efforts to contain corruption often fail. In the last two decades, however, studies have found a strong association between the share of women in elected office and lower levels of perceived corruption (Dollar et al., 2001; Esarey & Chirillo, 2013; Esarey & Schwindt-Bayer, 2018; Swamy et al., 2001). While equal opportunities and women’s rights are firmly rooted in human rights norms and declarations, the interest in women’s representation has gained substantial leverage from the notion that women representation could also change politics for the better (Lawless, 2004; Mansbridge, 1999; Phillips, 1998). International organizations, aid agencies and governments promote women representation as a cure for mismanagement, corruption and public service delivery failures. This has sparked an intense debate about how the share of women in politics is linked to lower levels of corruption.

Despite the strong and significant statistical association between women in office and lower levels of corruption, recent studies note that we know less about why and when this association occurs, and if it will last over time. Theoretical explanations for this link differ in directionality. While plausible theoretical frameworks and evidence suggest that women cause reductions in corruption (see i.e., Barnes & Beaulieu, 2018; Bauhr et al., 2019; Esarey & Schwindt-Bayer, 2018; Stensöta et al., 2015). studies also propose that low corrupt systems may facilitate the recruitment of women into office (Bjarnegård, 2013; Sundström & Wängnereud, 2016), or that underlying factors, such as the development of liberal democracy, may drive both more inclusive representation and lower level of corruption (Sung, 2003). Furthermore, recent studies note that the link between women representation and lower level corruption is context dependent, and the effects of women representatives may therefore differ depending on the positions and platforms that women gain access to, and thereby potentially also vary over time.

This study seeks to contribute towards addressing these difficult questions. We suggest that women mayors cause a reduction in corruption levels, but that the effects may not last over time. Thus, our study provides causal evidence in support for the rich theoretical frameworks suggesting that women in elected office reduce corruption, and suggests, in particular, that the gender of the mayor matters. However, we also suggest that in the highly competitive environment of assuming executive office, the women that adapt to corrupt networks over time are more likely to survive in office. We support our claims using quasi-experimental regression discontinuity design (RDD) and first difference (FD) estimation. Our evidence is based on unique and newly collected election data on municipalities over the past three electoral cycles matched with data on corruption risks in close to all major public procurement contracts awarded at the local level for French municipalities from 2005 to 2016. Our results show a clear and consistent reduction in corruption risks in municipalities where women mayors gain office. However, this effect is largely driven by newly elected women mayors. We find in sub-sample analysis that gender differences are negligible in municipalities where women mayor incumbents are re-elected.

We thereby make several important contributions to the literature. Recent studies note that an overwhelmingly large share of the literature employs observational research designs, and that we know comparatively little about whether including women in elected office actually causes reductions in corruption and not the least if the effect will last over time. Our study adds to the few recent attempts to gain a closer understanding of whether the link between women representation and lower levels of corruption is indeed causal (Brollo & Trioiano, 2016; Correa Martinez & Jetter, 2016; Esarey & Schwindt-Bayer, 2019; Jha & Sarangi, 2018). We employ a regression discontinuity design, and first difference design and unique data that, we argue, overcomes several of the limitations of previous designs employed, and thereby provides an important complement to existing studies. Our data also contributes to investigating this relationship with greater precision than do many past studies. We use municipal level data, which allows us to naturally control for several institutional factors that may affect results. Furthermore, most studies to date rely on perceptions-based (and often times country-level) measures and it is not implausible that country experts rely on gender equality as a heuristic for their corruption ratings. Our measure of corruption risks is not perception- based, and therefore provides an important opportunity to triangulate extant studies with more objective data.

Furthermore, while most studies to date focus on the legislature, using measures such as the share of women in parliament, we study the executive and compare municipalities with women mayors with those run by male mayors. While a growing literature argue for the importance of the executive office for women’s’ descriptive, substantive and symbolic representation (see i.e., Bauer & Tremblay, 2011; O’Brien, 2015), the gender of the mayor can matter also for corruption levels (Brollo & Trioiano, 2016). Marginalization theories sometimes suggest that the reason why women have limited influence over policymaking, despite their presence, is that that they “hit a glass ceiling” and are excluded from accessing executive or top positions. Thus, our focus on elected mayors offer an opportunity to study the effects of the exercise of power among women that access it.

Finally, our study takes time in office into greater consideration than do previous studies, and suggest that this also contributes towards developing our understanding of why women in office cause reductions in corruption levels Building on the rich body of literature seeking to explain why women representation may cause reductions in corruption levels (see i.e., Barnes, 2016; Barnes & Beaulieu, 2018; Bauhr et al., 2019; Bjarnegård, 2013; Dollar et al., 2001; Esarey & Chirillo, 2013; Esarey & Schwindt-Bayer, 2018; Escobar-Lemmon & Taylor-Robinson, 2009; Heath et al., 2005; O’Brien, 2015; Schwindt-Bayer, 2010; Stensöta & Wängnerud, 2018; Swamy et al., 2001), we propose that theories explaining why women reduce corruption differ in the extent to which they attribute this effect to women on average being socialized or incentivized into having a stronger demand for anticorruption reforms (what we call endogenous theories) or if they, instead are simply prevented from participating in corrupt transaction because of their marginalization and exclusion from elite networks (exogenous theories). Although directly studying why women in executive office reduce corruption is very difficult, the observable implications of these theories are likely to differ. If women reduce corruption because they carry a more endogenous demand for implementing anticorruption reforms, we might expect a status quo or perhaps even an increase in the effectiveness of women in reducing corruption over time, as they become increasingly networked and skilled in navigating in the political system. If exogenous theories, such as marginalization and exclusion from elite networks explain why women in office reduce corruption, we would expect, instead that the beneficial effect will subsequently diminish, as women gain increased access to networks or build their own collusive networks over time. The evidence in our study suggests that newly elected women mayors drive gender differences in corruption levels, while effects are less consistent among incumbents that are re-elected. One possible interpretation of our findings is thus that mayors that adapt to these political realities and manage to be included in the male dominated collusive networks where corrupt transactions are made, also survive in office.

## Are Women Politicians Less Corrupt Than Men?

In the last two decades, studies have consistently found a strong association between the share of women in elected office and lower levels of corruption (Alexander, forthcoming; Bauhr et al., 2019; Dollar et al., 2001; Esarey & Chirillo, 2013; Esarey & Schwindt-Bayer, 2019; Swamy et al., 2001; Watson & Moreland, 2014). While international organizations, donors and policymakers have been eager to incorporate this beneficial effect in their motivation as to why the share of women in elected office should be increased around the world (Towns, 2010, p. 158; Transparency International, 2016; UN, 2017), we know less about how this effect occurs, and in particular if it will last over time.

Recent studies raise the issue of directionality. In particular, we know comparatively little about whether including women in elected office actually causes reductions in corruption or if, instead, low corrupt and more meritocratic recruitment allows more women to assume office. Bjarnegård (2013) finds compelling qualitative evidence from Thailand that clientelism and network based recruitment benefit already privileged men at the expense of others, in particular women. Similarly, although on the basis of associational data from European municipalities, Sundström & Wängnerud (2016, p. 355) argue that the recruitment of women is more difficult in clientelist or corrupt systems because women are more likely to be excluded from the male dominated networks from which candidates are selected. In other words, “shadowy arrangements” benefit the already privileged, which in most contexts also tend to be men (Bjarnegård, 2013; Stockemer, 2011; Stockemer & Sundström, 2019).

The main problems with drawing causal inferences in this field have thus been unobserved heterogeneity in units of comparison along with non-random selection into the “treatment,” which introduce issues of endogeneity. To address this issue, a few studies (Correa Martinez & Jetter, 2016; Esarey & Schwindt-Bayer, 2019; Jha & Sarangi, 2018) have not only theoretically but also empirically addressed the problem of endogeneity using an instrumental variable approach.^1^ Esarey and Schwindt-Bayer (2019) conclude that causality runs in both directions: women’s representation decreases corruption, and that corruption decreases women’s participation in government. The RD design provides an important and thus far underused complement to this approach since it is not dependent upon finding valid instruments, while maintaining a potentially higher external validity compared to lab experiments.

Closely investigating whether this effect is indeed causal is important not the least since improving women’s representation is generally regarded as being comparatively responsive to political and institutional manipulation. While decades of corruption research have identified several factors that may cause lower levels of corruption, including certain colonial origins or geographical location, few of them are actually implementable policy reforms (Rothstein, 2018). However, in order to understand this effect we need to carefully delineate in what roles women in political office can make a difference. Women mayors in advanced democracies such as France should stand a strong chance to affect the direction of political processes. This leads to our first hypothesis.

H1. The inclusion of women in executive office causes a reduction in corruption levels.

Several plausible theories on why there is an association between women and corruption has been developed over the last few decades. Early studies on the link between women’s representation and corruption note that women are socialized into being more honest and trustworthy than men (Dollar et al., 2001). Women have been found to be more pro social than men, and thereby more likely to engage in “helping” behavior and to base voting decisions on social concerns on average (Eagly & Crowley, 1986; Goertzel, 1983). Several studies have directed particular attention to the notion of women being more risk averse than men (Esarey & Schwindt-Bayer, 2018; Swamy et al., 2001). Studies from a variety of different fields suggest that women are typically seen as more risk averse than men (Booth & Nolen, 2012; Bord & O’Connor, 1997; Flynn et al., 1994; Huddy & Terkildsen, 1993; Slovic, 1999; Watson & McNaughton, 2007). Studies also suggest that women politicians are sometimes perceived as more honest than men (Barnes & Beaulieu, 2014, 2018) and thereby also more severely punished for engaging in corruption by the electorate, which de facto increases the risk for women engaging in corruption (Eggers et al., 2018; Esarey & Schwindt-Bayer, 2018, p. 5). However, there may be reasons to expect women in elected office and perhaps particularly women in executive roles to be less risk averse than women on average since evidence suggest that the gender gap in risk aversion is substantially smaller among elites (Lapuente & Suzuki, 2020). Furthermore, mobilizing against corruption can also be extremely risky, and therefore not necessarily an attractive war to wage for risk averse actors, which suggests the need for complementing theories.

Building on the previous theories on pro sociality, risk aversion and electoral expectations, studies also suggest that the beneficial effect of including women in elected office may be attributed to women politicians having a different political agenda than men (Bauhr et al., 2019; Lawless, 2015). Women politicians are on average more likely to prioritize the improvement of public service delivery, and in particular the type of services that primarily benefit women (Bolzendahl, 2009; Bratton & Ray, 2002; Dolan, 2010; Ennser-Jedenastik, 2017; Jha & Sarangi, 2018; Schwindt-Bayer & Mishler, 2005; Smith, 2014). Furthermore, women may mobilize against corruption in order to break collusive and corrupt male dominated networks that are detrimental to their political careers (Bjarnegård, 2013; Goetz, 2007; Stockemer, 2011; Sundström & Wängnerud, 2016). Both of these political agendas contribute towards making women prioritize the fight against corruption to a greater extent than men.

Thus, influential theories to date suggest that demand for anticorruption could be seen as endogenous to women representatives since women are socialization into particular norms or incentivized to demand cleaner government. A different perspective emerges from work on explanations that are primarily exogenous to women representatives, and pertain instead to women’s opportunities to participate in corrupt transactions. These explanations focus less on women’s pro sociality, risk aversion or political agendas, and more on women being marginalized and thereby excluded from the tightly knit networks where corrupt transactions are made (Barnes, 2016; Barnes & Beaulieu, 2018; Bjarnegård, 2013; Escobar-Lemmon & Taylor-Robinson, 2009; Heath et al., 2005; O’Brien, 2015; Schwindt-Bayer, 2010). Insiders often benefit from corrupt transactions, while political outsiders are typically excluded from their benefits (Bauhr & Charron, 2018), and women candidates are often depicted as a political outsider (Carpini & Fuchs, 1993; Dolan, 1998).

Directly investigating why women in elected office reduce corruption is difficult. However, the observable implications of endogenous theories are partly different from those of exogenous theories. In particular, if women reduce corruption primarily motivated by endogenous factors, that is, because they (or their constituents) demand such change, reductions in corruption should be unrelated to their level of seniority in office. If anything, we could expect women mayors to become increasingly skilled and effective in reducing corruption as their network and influence expand. If, on the other hand, women in elected office reduce corruption because they were prevented from participating by primarily exogenous factors, and in particular because they are less networked newcomers to politics with limited access to collusive networks, we would expect the corruption reducing effect of including women in office to subside over time. As women grow more senior in their executive role, we would expect corruption to return to more normal levels. Women may develop their own networks over time, and these networks are not necessarily less corrupt. We suggest here that women who not only attain office but also remain in office for an extended period of time may be more likely to adapt to the current rules of the game, build their own networks and not necessarily reduce corruption levels over time (Goetz, 2007, p. 95). This in turn could be explained both by selection effects (i.e., only women that adapt to corrupt networks manage to survive in executive office) and an adaptation effect (meaning that women adapt to politics as usual over time). This forms our second hypothesis.

H2. Compared with men, newly elected women mayors reduce corruption, while incumbents that are re-elected have limited effects on corruption levels.

## The French Case

Our analysis employs data from a sub-set of the roughly 36,000 municipal elections from the past three cycles in France. Studying the effects of women’s representation on corruption in French local elections has several advantages. In general, the sub-national level of analysis potentially provides more validity in comparing across cases, as many of the confounding factors that vary across countries are controlled for “naturally” (Snyder, 2001), thus avoiding potentially spurious effects of such institutions as media freedom, democracy, rule of law etc. (Sung, 2003). France is an exceptional test case for this since the sheer number of municipalities in France is larger than in any other European country, offering a greater number of observations. Surprisingly, France is a relatively understudied country in the corruption literature, with many single country studies focusing on the U.S., Spain, U.K., Brazil or Italy, and our study thereby adds much needed insight about this case. A further advantage of the French case is that France initiated gender quotas in a 2000 law (law 2000-493), whereby party lists had to present gender balance for elections with a proportional representation system, meaning that the parity system is applied for municipal, legislative, regional, senate and European elections for a certain population (3,500 inhabitants in 2008 and over 1,000 from 2014), which applied for the first time to the 2001 election.^2^ This has led to the steady rise of the share of women in local politics.^3^ The party lists has led to more or less gender balanced municipality council in localities over the population threshold; municipality councils have between just under or over 50% female representation depending on the list order and number of seats.^4^ Thus, any gender difference we observe is isolated to the office of the mayor. Finally, the mandate of a French mayor allocates them considerable power and discretion over certain policy areas and thus makes for a relevant unit of comparison. We provide more information on French municipalities and mayors in Supplemental Appendix 6.

## Design, Sample, and Estimation

The main goal of our empirical analysis is to advance our understanding of any causal effects of gender on corruption. Most previous comparative studies use comparative observational research designs, thereby facing potential endogeneity bias. The main methodological challenge is that polities where the support for women politicians is high enough to result in the election of a women executive may differ systematically from polities where the support for women is more tenuous and results in the election of a male executive.

To complement existing studies, we elucidate greater causal inference primarily via a regression discontinuity design (RDD) using the local level (municipality) as the unit of analysis. In this design, the cases above a pre-determined threshold in an observed independent variable receive the treatment. The RDD has been employed to assess the effects of close electoral outcomes on a wide scope of socio-political outcomes (for example, Brollo & Troiano, 2016; Eggers et al., 2015; Klašnja & Titunik, 2017; Lee et al., 2004). Using an RD design to test our hypotheses offers several advantage over previous designs. It avoids the difficulty of finding a truly exogenous instrument that is associated with women’s representation but not with corruption (perceptions). The data and design also often offers a generally higher degree of external validity compared to lab-experimental approaches that rely on hypothetical scenarios and sometimes also student samples (cf Alatas et al., 2009; Rivas, 2013). Thus, the design offers new opportunities to study this relationship over time and in different settings (Brollo & Trioiano, 2016).

Essentially, we would like to estimate the difference in corruption when our unit of analysis (municipalities) “i” is run by a woman $C_{i}\left(1\right)$ as opposed to one run by a man $C_{i}\left(0\right)$. The problem of causal inference in our case is that we cannot observe both outcomes simultaneously. Since gender randomization of electoral success is out of our control, an experimental design is not an option, and thus RD offers us the design with the greatest level of causal inference. Similar to Brollo and Troiano (2016), we elect to match cases in mixed gender elections, where the two leading candidates are a man versus a woman, and thus observations present the possibility of either winning a given election.

Given that the data meet the proper assumptions of this design, the municipalities with close, mixed gender elections are then assumed to be more validly comparable, which is known as the local effect, $\left(M_{i}\in \left[-h,h\right]\right)$, meaning that a man winning under similar conditions constitutes a valid counterfactual.^5^ M is the margin of victory for the women candidate, which will be a positive number when a woman is successful and negative if otherwise (where “0” is the threshold value). We define this parameter as the vote share for the women candidate’s list minus the vote share of the party list led by a male candidate,^6^ and as M (the running variable) is determinative of the treatment, we employ a sharp RD.

Since the female margin of victory can be correlated with unobserved factors in the model, $e_{i}$, we attempt to remedy the issue of endogenity with a non-parametric, local linear regression and via fitting a p-order polynomial in $M_{it}$ on both sides of the threshold (e.g., when $M_{i}=0)$.^7^ The local linear regression thus limits our sample to those cases that are sufficiently close to the threshold (Imbens & Lemieux, 2008),and to avoid arbitrary choices of the bandwidth “h,” we employ the data driven method established by Calonico et al. (2014).The estimand of interest is then the local average treatment effect “τ” (LATE), for example, levels of corruption in male led municipalities vis-à-vis women led ones at the threshold (M = 0); estimated as follows (from de la Cuesta & Imai, 2016):

(1) $$\tau =E\left(C_{i}^{1}-C_{i}^{0}|M=0\right)=\underset{M↓0}{\lim}E\left(C_{i}|M\right)-\underset{M↑0}{\lim}E\left(C_{i}|M\right)$$

In addition to the RD estimates, we exploit the time dimension of our data in a sub-sample of municipalities for which we observe a change in mayor at some time during the period. For this, we code municipalities into one of four possible groups—(1) a change in mayor from one male to another male, (2) a male to a female, (3) a female to a male and (4) a female to another female. We compare average changes in corruption risks within municipalities over time via a first difference (FD) estimator, accounting for control variables and municipal-level clustered standard errors.

We focus on municipalities with the closed list proportional representation (PR) system—that is to say municipalities over 3,500 inhabitants in 2008 and those over 1,000 in 2014 (see Supplemental Appendix 6). Election results data are from 2008 and 2014 taken from official French election sources, published by the Ministère de l’Intérieur.^8^ We found that, in 2014, 1,863 municipalities had a mixed gender election that meets our criteria, whereas 671 had such an election in 2008. Data from with 2001 election were also gathered for purposes of comparing the 2008 sub-set with the previous mandate period (lagged effects, etc.).

## Variables

### Dependent Variable

Despite its growing interest among academics and policymakers alike, the concept of corruption remains an elusive and challenging one to measure (Klitgaard, 1988). We use the common definition of corruption as “private gain at the public’s expense” and, in this case, we are most interested in capturing elite, political corruption at the local level. The often used perception based measures are unlikely to allow us to precisely test our hypotheses as their validity can even be questionable, in particular since some respondents might use gender equality as a heuristic for lower corruption. Moreover, our level of analysis (municipalities) is at a lower level than standard measures of corruption, which tend to capture either the country or provincial level (Charron et al., 2015), thus there are limited data options at this level of analysis. Therefore, we elect to proxy the level of corruption in a municipality by taking advantage of recently released objective data on corruption risks in public procurement (Fazekas & Kocis, 2017). While some might argue that France is a relatively low corrupt country,^9^ corruption in procurement, unlike petty corruption, occurs at a high (often unseen) level and plagues both developed and developing countries Bauhr et al. (2020).^10^ In addition to a large number of observations in terms of municipalities, France is also a case with the greatest amount of procurement contracts in the dataset—with 1.08 million available from, 2005 to 2016. In terms of economic significance, local procurement accounts for roughly 6% of total GDP in France,^11^ which in 2013 budget terms for example, constituted roughly 117 billion Euros.

The data on public procurement contracts for France during the 2005 to 2016 period provide information on “red flags” for example, warning signs that high level collusion is more likely.^12^ As most of the red flag items proxy transparency rather than corruption (Bauhr et al., 2019), we employ the measure closest to our concept of corruption: the proportion of single bidding in a municipality, which is the indicator that is also most widely available across French municipalities. This objective measure that taps into the deliberate restriction of competition in order to favor well-connected firms to politicians and has been used in several recent studies to proxy for high level corruption (Bauhr et al., 2019; Charron et al., 2017; Fazekas & Kosics, 2017). In addition, the data specify what level of government to which the contracts are assigned—EU, national or local. For the sake of precision, we aggregate the data annually for each contract that is labeled as a local level procurement, and municipal mayors ultimately authorize all projects in their jurisdiction.^13^ As the contracts are all geo-coded to the municipal level, we then matched all municipal corruption risk data with our sample on mixed gender elections. The data are available from 2005 to 2016 and we therefore have access to several years prior to and following both the 2008 and 2014 elections. To maximize our observations, the yearly data are aggregated for the entire mandate period.^14^ For the 2008 election, the data are for six years—2008 to 2013; while data for the 2014 mandate period are from 2014-2016. We also check for any systematic differences in male versus female led municipalities prior to the mandate period in question via a lagged dependent variable (Eggers et al., 2015), whereby we find no significant differences (see Supplemental Figures A4 and A5 in the Appendix)

A weakness of this measure is that while objective, it is clearly not a direct measure of corruption per se. As no perceptions or citizen-based petty-corruption measure exists at this level, an alternative to our measure might be reported corruption scandals in the media (Costas-Pérez et al., 2012). However, as many municipalities lack local media oversight, this measure would most likely overlook “actual’ corruption in many smaller areas and introduce endogeneity, as municipalities in which mayoral corruption is actually detected and reported are likely systematically different from places where it is not. Thus, we argue that our measure is the best available proxy.

### Independent Variables

The sorting variable in this analysis is the margin of victory of the woman mayoral candidate in a given election. The threshold is normalized at “0,” and municipalities have a value between −1 and 1; thus any positive values represent a female victory (and “treatment,” heretofore called “female win”) and negative values represent a male victor (and “control”). Supplemental Figure A1 in Appendix 2 shows a sample wide scatter plot of the two main variables. We observe that there is a slightly higher proportion of single bidding among male-led municipalities (0.23) than women-led ones (0.20) for the sample on the whole.

### Control Variables

At the municipal level, we collected a battery of possible confounding socio-economic factors that are highlighted in the literature. First, we take the level of economic development as a measure of monthly income per capita. Second, we account for the level of income inequality via the ratio of the average management salary (“cadres”) over the average worker. Higher ratios equate to greater inequality. Third, we proxy the level of education in a locality with the percentage of residents with higher (tertiary) education. Fourth, we capture the strength of the labor market via the unemployment rate at the start year of the mandate period. Fifth, as many of the red flags, such as single bidding, might simply be a function of the lack of competitiveness of the local market, we control for the number of total and commercial only registered firms in each municipality, as well as the overall population and population density.

At the election level, we control for the number of parties competing in each municipality as well as the number of rounds (one or two), along with the turnout in each round where applicable. As per information on the mayors, we have no reason to suspect that one party is any more corrupt than another in this context; thus we take a parsimonious measure of party alignment—whether the mayor’s party aligns with that of the sitting president.^15^ We also take the age and previous occupation of the mayors themselves. As our second hypothesis highlights the “newcomer effect,” we code whether the mayor is an incumbent or not. An additional confounding factor could be the overall gender composition of the local councils. However, as noted, the councils have ‘built-in’ gender balance due to the quota system, the gender variation is isolated at the mayoral level. Next, we account for whether one group received more (or less) total procurement contracts during their mandate period. Finally, the year is included, as 2014 marked a year in which the threshold for the list system was changed from 3,500 to 1,000 inhabitants. In Table A1 we observe the means for the sample as a whole as well as those of male and female led municipalities respectively. We find that, in general, the two sides are quite well-matched, yet with several exceptions in our difference of means tests. Further summary information on data and sources can be found in the Supplemental Appendix, section 2.

### Assumptions of the Design and Threats to Validity

Researchers commonly posit the “as if random” assumption when employing an RD design. This is to say that, within a given range (“bandwidth”) on either side of the threshold of one’s sorting variable, receiving the treatment can be considered as being randomly assigned (Lee et al., 2004). This relies on the stringent assumption that observations on either side of the threshold within this given range are indistinguishable with respect to confounding variables. However, as several recent studies have argued, the validity of the design still holds under the weaker ‘continuity assumption’, which is to say that “that the only change, which occurs at the point of discontinuity, is the shift in the treatment status” (de la Cuesta & Imai, 2016, p. 377). Additional threats to the design’s key assumption also have to do with unit sorting of observations near the threshold, in that candidates or parties may be manipulating results in some way prior to or after the election (Eggers et al., 2015). We test these assumptions empirically (Supplemental Appendix, section 3).

While there are many testable potential violations to the design’s assumptions, there are some that are also untestable given a current lack of data. One, H1 could be driven by a strategic nomination process, where parties nominate certain types of candidates in safer seats compared with nominations for more competitive seats, thus to interpret our findings, we must make the assumption that this difference is negligible, or unassociated with corruption levels.^16^

In sum, while we of course cannot account for all possible confounding effects, among those that we do observe directly, we do not find any obvious threats to the validity in our design. We proceed to the results in the following section.

## Results

**Figure 1.**
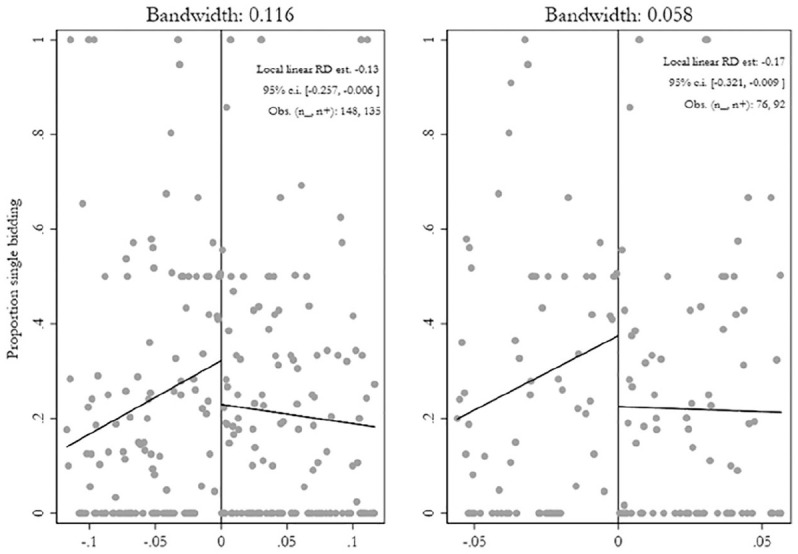
Female margin of victory and corruption risks: RD plot. *Note*. RD plot with all observations on either side of the threshold fit with a first-polynomial order (linear). The *x*-axis shows the female win running variable with female mayors on the positive side and male on the negative side. The risk for high level corruption is on the y-axis, measured as the proportion of contracts that are single bid per municipality-mandate period. Bandwidths are determined from via coverage error rate (CER) with the aid of the data driven algorithm of Calonico et al. (2014), and are taken from models 2 and 3 in Table 1.

Table 1 presents the OLS and RD estimates for the whole sample (H1). Looking at the OLS results in column 1, we find that the marginal effect of women mayors indicates that they have slightly less corruption risk (3.4% less) than males on average, and the one-tailed significance test shows that the difference is significant at the standard 95% confidence level. However, column 2 shows an RD roughly 3.5 greater than the OLS estimate: −0.14, or 14% less single bidding (equal to roughly 50% of one standard deviation of the dependent variable). This result from model 2 using the first-order polynomial shows a similar estimate as in Figure 1, in which the fourth polynomial was used. We reduce the suggested bandwidth by one-half (0.057) in column 3, resulting in an even larger gender effect (−0.20). We double the selected bandwidth in column four, where we observe similar effects to models 2. Columns 5 and 6 employ local linear regression using higher level polynomials, which both show evidence in favor of female mayors lowering corruption risks. The results seen in column 2 are supported via these estimations.

**Table 1.** The Effect of Gender on Corruption Risks: RD Estimates.

Dep variable	Single bidding				Quadradic	Cubic
Control function	None	Linear				
Bandwidth	Global	H	h/2	2/h	h	h
	1	2	3	4	5	6
Female mayor	−0.034	−0.14**	−0.20**	−0.14**	−0.18**	−0.19**
95% C.I. bias corrected	[−0.072, 0.004]	[−0.265, −0.017]	[−0.360, −0.047]	[−0.234, −0.041]	[−0.332, −0.027]	[−0.365, −0.016]
95% C.I. robust	[−0.071, 0.003]	[−0.275, −0.007]	[−0.390, −0.017]	[−0.269, −0.006]	[−0.336, −0.023]	[−0.368, −0.013]
Bandwidth	1.00	0.116	0.058	0.228	0.132	0.158
R^2^	0.04					
Total effective obs.	866	283	168	499	316	358
Control obs.	541	148	76	262	150	172
Treament obs.	325	135	92	237	166	186
Mean single bidding	0.219	0.222	0.241	0.232	0.222	0.222

*Note*. Column 1 displays results from simple OLS regression, while columns 2-4 are local linear regressions with first-order (linear) polynomials. Columns 5 and 6 use quadratic and cubic polynomials, respectively. The running variable is the margin of female mayoral victory with the sharp cut-off at “0” and a range of −1 to 1. Bandwidth (“h”) is determined via coverage error rate (CER) with the aid of the data driven algorithm of Calonico et al. (2014), and all local linear regressions use triangular (e.g., weighted) kernel functions, such that observations closer to the threshold have greater weight values. Significance determined by the 95% confidence interval of the estimate, with the second confidence interval clustered by municipality. The number of observations is shown in total as well as in the number of observations on either side of the cut-off, with male mayors representing the control and female representing the treatment groups. The mean of the dependent variable in each model is presented in the final row.

*** *p* < .01, **p < .05.

Figure 1 presents summary visuals of the main results in Table 1, highlighting models 2 and 3. The main point of interest is the gap at the threshold, which we hypothesize, is significantly higher for male mayors (left side). We observe that the local linear effect with the recommended bandwidth (a margin of victory within 11.7%) indicate 13% less single bidding among women-led municipalities, while it is 17% less when we reduce the bandwidth by half (a margin of victory within 5.8%).

**Figure 2.**
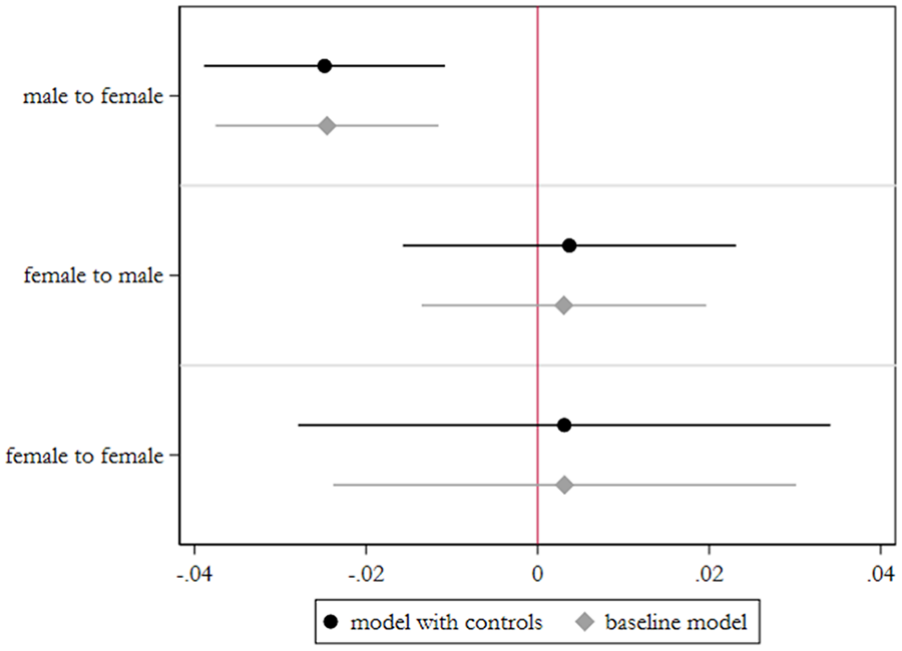
Difference in corruption risks in municipalities with mayoral change. *Note*. First difference estimates shown with 95% confidence intervals, clustered by municipality. Reference group is male to male. Sample includes only municipalities with a change in mayor during the observed time period. Full table, A4, is given in the Supplemental Appendix 5.

As a complement to the RD estimates, we exploit the variation over time within municipalities for which we observe a change in mayor during the period in the analysis. According to H1, we should expect that corruption risks decrease in a municipality at the highest rate among those that changed from male to female leadership. To assess this, we estimate the first difference (FD) of corruption risks in each group,^17^ using the change in male to male mayor as the reference group. Figure 2 summarizes the findings. We observe that, indeed, among those municipalities where we observe a change in mayor, those that went from a male mayor to a female mayor on average yield the greatest decreases in corruption risks—a difference of roughly 2.5 less single bidding on average compared with municipalities that changed from male to male. The female to female yields no significant difference from the male to male group. Interestingly, the coefficient for the female to male group is statistically negligible as well. While much of the literature theorizes on the effects of increases in gender equality on corruption, few to our knowledge posit a theoretical expectation on the reverse direction—what happens to corruption when a female-led polity becomes male-led again. While we do observe a small increase in corruption level in this group, the insignificance could be due to the limited sample size (there are fare fewer past female incumbents), or possibly due to the most corrupt, worst performers being voted out. The results are robust to an addition of all control variables in Table 1. In sum, both RD and FD estimates point to evidence for H1 that going from male to female led municipalities decrease corruption risks.

## Test of Hypothesis 2

To test H2, which refers to the time in office, we test heterogeneous effects of gender on corruption risks for split samples—incumbents and newly elected mayors.^18^ The assumption here is that incumbents have had at least 6 years (if not more) to build local networks, while newly elected mayors are less likely to have done so. In these analyses, the estimand of interest is the conditional local average treatment effects (“CLATE,” Hsu & Shen, 2019):

(2) $$\mathrm{CLATE}=E\left[Y_{i}\left(1\right)-Y_{i}\left(0\right)|I_{i}=x,\;M_{i}=0\right]$$

Where the average treatment effects are estimated at the threshold (“0”), conditioned by values of I (incumbency or not). In this case, the running variable (margin of victory in election t), is related to past values of the conditional variable (I), in that incumbency is “1” only if $M_{t-1}>0$. For incumbents (I = 1) in particular, this implies that the counterfactual to an incumbent female is an incumbent male, which is not realistic and introduces potentially unobserved confounding factors. We therefore elect to look at incumbency in women candidates only who run against males (sample one) and the effects of the newly elected women running against males (sample two), and then compare these two RD estimates. Thus, while the treatment is determined differently in the two samples (sample one requires that I = 1 and M > 0, while in sample two, I = 0 and M > 0), the counterfactual is consistent. If H2 is corroborated, we should observe negative and significant effects for the latter group, but not the former.

**Figure 3.**
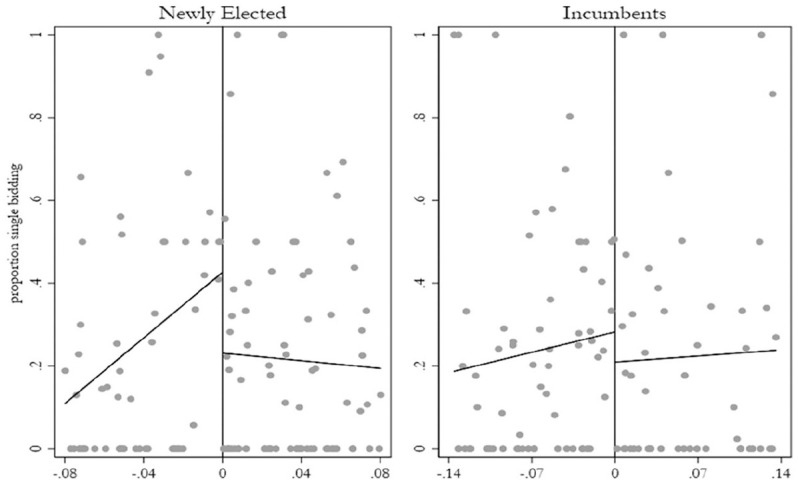
Separate RD plots of newly elected and incumbent mayors. *Note*. RD plot with equally spaced bins on either side of the threshold fit with a first-polynomial order (linear) from column 2 in Table 2. The *x*-axis shows the female win running variable with female mayors on the positive side and the male on the negative side with all observations within the recommended bandwidths. The risk for high-level corruption is on the *y*-axis, measured as the proportion of contracts that are single bid per municipality-mandate period.

In group two however (municipalities with newly elected female mayors), we observe a large and consistently significant gender gap, which would suggest support for H2—that the gender gap in corruption risk that we observe is driven by newly elected women leaders. While the estimate of the local linear regressions varies depending on the bandwidth and polynomial order, the finding is that newly elected women demonstrate between 0.15 and 0.26 (or 15%–26%) less single bidding compared elected males—the latter of which constitutes roughly one standard deviation of the dependent variable. Moreover, the RD effect is observed to be greater the smaller the bandwidth used. In the first group (municipalities with incumbent females), we still observe lower levels of corruption risk among the women mayors on average, although none of the differences in any of the model specifications are statistically significant. Moreover, the RD effects become smaller as the bandwidth tightens. In the last row, we formally test the differences in these coefficients with a two-sample t-test. We find mixed results, with the coefficient for newly elected females being significantly greater than the effect of incumbents in model 3 and 4, but not in others, particularly in model 2 with the recommended bandwidth. RD plots summarizing the main findings of Table 2 are in Figure 3.

**Table 2.** The Effect of Gender and Time in Office on Corruption Risks: RD Estimates.

Dep variable	Single bidding				Quadradic	Cubic
Control function	None	Linear				
Bandwidth	All mixed-municipalities	h	h/2	2/h	h	H
	1	2	3	4	5	6
Group 1: incumbent female mayors						
Female mayor	−0.046	−0.151	−0.079	−0.134	−0.177	−0.204
95% C.I. robust	[−0.131, 0.037]	[−0.366, 0.057]	[−0.564, 0.406]	[−0.338, 0.068]	[−0.413, 0.058]	[−0.543, 0.139]
Bandwidth	1.00	0.111	0.055	0.222	0.186	0.165
Effective Obs.	648	175	99	328	266	238
Control Obs.	520	135	74	254	204	180
Treatment Obs.	227	42	25	74	62	58
Mean single bidding	0.222	0.229	0.247	0.212	0.219	0.243
Group 2: new female mayors						
Female mayor	−0.049	−0.153***	−0.261***	−0.148**	−0.179**	−0.180**
95% C.I. robust	[−0.132, 0.033]	[−0.298, −0.008]	[−0.455, −0.068]	[−0.292, −0.004]	[−0.348, −0.011]	[−0.359, −0.001]
Bandwidth	1.00	0.104	0.052	0.208	0.124	0.169
Effective obs.	747	229	120	392	257	325
Control obs.	520	129	62	241	143	187
Treatment obs.	227	100	58	151	114	138
Mean single bidding	0.223	0.230	0.257	0.232	0.229	0.234
Test of difference in coefficients between new females incumbent females						
T-test (*p*-value)	.08	.41	.000	.02	.45	.97

*Note*. The running variable is the female mayor’s margin of victory at *t*, the dependent variable is risk of corruption in public procurement during the mayor’s full electoral mandate period. Treatment in the first set of estimates is newly elected mayors, and incumbent female mayors are the treatment group in the second set. All municipalities run by males serve as the control group. In column 1, we report the average marginal effect via OLS. In columns 2–6, the estimate reported is the conditional local average treatment effect (CLATE) via local linear regression with triangular kernel and CERRD optimal bandwidth. Columns 2–6 report the 95% robust confidence interval with standard errors clustered on the municipality, main optimal bandwidth, total effective observations, treated observations within bandwidth, and control observations within bandwidth (*represent 1-tailed test). The *t*-test in the bottom panel summarizes a t-test of differences in coefficients across the two samples, testing whether the effect of new mayors (“n”) is significantly different from incumbents (“i”), with the formula: $\beta_{n}-\beta_{i}/\sqrt{SE_{n}+SE_{i}}$. As H2 is directional, the *p*-value reported is one-tailed.

**Figure 4.**
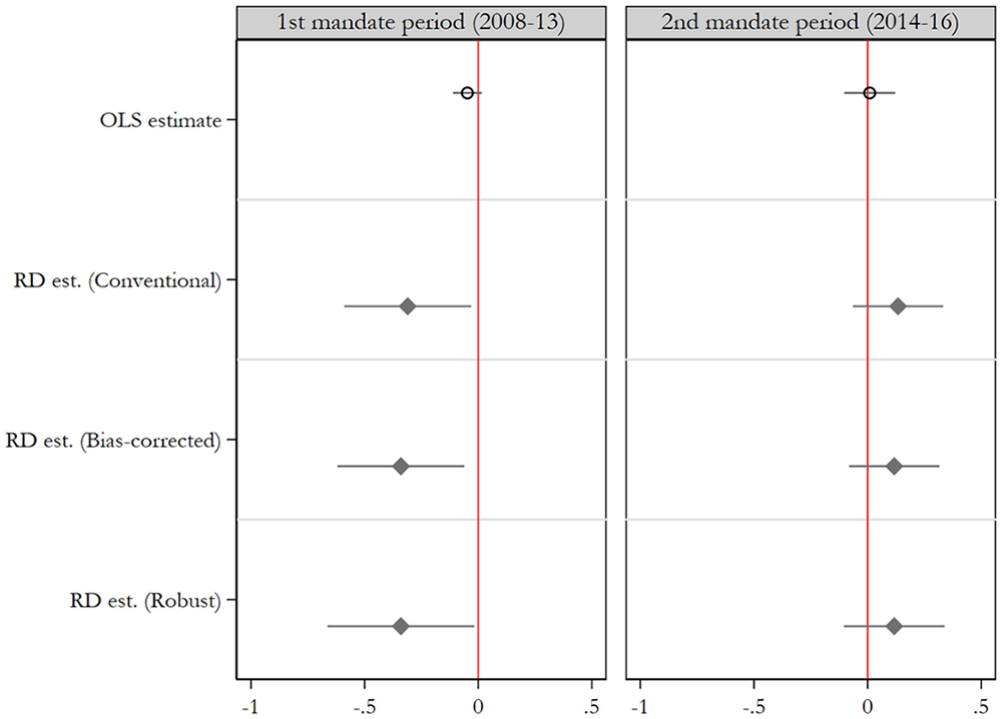
Gender gaps in corruption risks in 1st and 2nd mandate periods among newly elected mayors in 2008. *Note*. OLS estimate includes all relevant cases (e.g., bandwidth = 1), while the RD estimate reported is the local average treatment effect via local linear regression with triangular kernel and CERRD optimal bandwidth, which is 0.094 and 0.126 for the left and right columns, respectively. See Supplemental Appendix 5, Table A5 for full tables.

Finally, we compare a sub-sample of newly elected mayors from 2008 to test whether the gender gap in corruption remains among the same mayors over multiple mandate periods. Figure 4 highlights the results of OLS and RD estimations. In the first column, similar to Table 2 (but excluding newly elected from 2014), we show the effect of newly elected women mayors compared with males, which shows a negative and significant effect for several different RD estimates. However, comparing the gap among the same set of mayors who also won re-election in 2014, we find that women mayors have higher corruption risks than males in their second mandate period, although the effect is not significant.

Similar to the results given in Table 2, the results show that women that are reelected are less likely to reduce corruption risks. This suggests that the beneficial effect of women in office is reduced over time, and that women mayors reduce corruption in particular because of their marginalization and exclusion from elite networks. If women reduce the risk for political corruption because of factors that are largely exogenous to them, their opportunities to participate in corrupt transactions may increase over time, at least among those women that manage to navigate the system and become reelected. If women politicians would primarily be motivated by a more endogenous demand for lower levels of corruption, we would expect the opposite relationship, that is, that women would grow more skilled at pushing their anticorruption agenda as their time in office and experience increases.

## Additional Checks for Alternative Specifications

As there are many choices on the estimates imposed by the researcher in the RD design, we re-run the results of Tables 1 and 2 using three researcher selected bandwidths (0.25, 0.10, and 0.05) along with four different polynomial orders (first to fourth-order). We test various data driven bandwidth approaches as well as include control variables in the models. We find the main results to be robust to these alterations. The results can be found in the Supplemental Appendix (section 4). In addition, we tested whether Table 2 was robust to the inclusion of control variables and find that the results hold (see Supplemental Appendix 4, Table A2).

## Discussion and Conclusion

This study investigates the difficult question of whether women in executive office cause reductions in corruption levels and, if such effects will last over time. Using a regression discontinuity design and unique municipal level electoral data, drawing from roughly 36,000 municipal elections in France together with corruption risk data on close to all major public procurement contracts awarded during 2005 to 2016, our results show that women mayors reduce corruption risks. However, our results are largely driven by newly elected women mayors, and we do not observe clear gender differences in corruption risks in municipalities where women incumbents are re-elected.

Understanding the influence of women in politics is important in particular since policy makers, experts and international organizations motivate an increased women representation not only as a goal in its own right, but also as a means to make politics better. However, despite an impressive body of research showing a strong association between women representation and lower levels of corruption, studies question if the association is causal, raise the issue of, directionality and point to potentially confounding effects. This study adds to an emerging body of work that provides closer causal evidence for the beneficial effects of women in politics (set i.e., Brollo & Trioiano, 2016; Correa Martinez & Jetter, 2016; Esarey & Schwindt-Bayer, 2019; Jha & Sarangi, 2018). With our design and unit of analysis, the gender effects are tested at a lower level of governance than many comparative studies (municipal) and via an objective measure of corruption risk of local procurement contracts, over which mayors are ultimately responsible. Our sample also permits comparison of gender effects of executives (mayors) rather than legislatures (percentage women parliamentarians), which lends our study added precision.

Furthermore, investigating not only if women representation reduce corruption but also if effects last over time allow for additional insights into why women representation reduce corruption. We propose that the rich body of work providing theoretical propositions for why women reduce corruption can be divided into two broad and admittedly diverse groups. The first group focuses on women being socialized or incentivized into having a stronger demand for anticorruption reforms (what we call endogenous theories). The second group suggests that women reduce corruption levels simply because they lack opportunities to participate in corrupt transactions, since they are excluded from the tightly knit and often-male dominated net works where such transactions take place (what we call exogenous theories). If newly elected women are more likely to reduce corruption risks, as suggested by this study, this may suggest support for marginalization theories, where women initially excluded from networks and opportunities gain such opportunities with seniority in office. More endogenous theories might suggest, instead, that women with seniority would grow increasingly skilled at pushing their anticorruption agenda. However, it is important to note that this study does not directly investigate why women reduce corruption, and other interpretation could be consistent with our findings. Furthermore, we do not know if women candidates that push to a radical anticorruption agenda simply fail to be re-elected, that is, if our results can be attributed to a selection effect rather than an adaptation effect.

Thus, we see a number of potential avenues for future research as data becomes available. Under what circumstances women reduce corruption and also whether this effect continues over time clearly warrants more attention, since it may be contingent on the positions women attain, and what kind of corruption they deem most salient and in what contexts (Bauhr & Charron, 2020). Our evidence relies on data from a subset of units in a single country with a relatively high proportion of female representation at the local level, and previous research suggests that the relationship between women’s representation and corruption is moderated by factors such as political systems (Esarey & Chirillo, 2013), thus we are cautious in terms of broad generalizability. It is also important to note that while our analysis adds empirical evidence for women mayors causing reduced corruption risks, our analysis does not in any way contradict recent findings that causality may also run in both directions (Esarey & Schwindt-Bayer, 2019) or that low corrupt systems may facilitate the inclusion of women in politics (Sundström & Wängnerud, 2016). Our results also point to the importance of closely investigating not only the women that attain executive power but also those that manage to retain it over time. However, providing causal evidence for the effect of women in executive roles is important for anyone interested in women’s’ role in anticorruption. These theoretical and empirical advances are important to understand the complex dynamics that surround the inclusion of women in elected office, and thereby if women executives will contain corruption in the short run, as well as continue to do so over time.

## Supplemental Material

sj-pdf-1-cps-10.1177_0010414020970218 – Supplemental material for Will Women Executives Reduce Corruption? Marginalization and Network Inclusion Click here for additional data file. Supplemental material, sj-pdf-1-cps-10.1177_0010414020970218 for Will Women Executives Reduce Corruption? Marginalization and Network Inclusion by Monika Bauhr and Nicholas Charron in Comparative Political Studies

## Notes

1 These studies use instruments such as the year of women’s suffrage and time since transition to agriculture, (Jha & Sarangi, 2018), plow use (Correa Martinez & Jetter, 2016) or lagged independent variables (Esarey & Schwindt-Bayer, 2019) as instruments.

2 While gender quotas apply to party lists, the gender of the head candidate is not regulated by the law.

3 For example, the number of women municipal councilors in 1995 was 21.7% and increased to 34.8% in 2008. The percentage of female mayors in the same period nearly doubled—from 7.5% in 1995 to 14% in 2008.

4 While it might be the case that some municipalities have more skewness toward male representation if all parties that obtain seats are led by males, in our sub-sample the gender balance is greater as either the first or second largest party is led by a female. The % of female councilors in our sample is between 45.5% and 55.5%, with the 5th and 95th percentiles of the sample being 48.1% and 51.8% respectively.

5 While there is some debate as to whether such elections are truly “random” due to incumbent party advantage, in particular in U.S. elections (Caughey & Sekhon, 2011), the findings of Eggers et al. show that incumbent advantage in close French municipal elections is non-significant (Eggers et al., 2015, pp. 267–269).

6 In the cases where there are more than two party lists, we look at the top two contending lists.

7 While earlier studies employed higher-order polynomials (Lee, 2008), there is some recent skepticism over whether they should be employed in RDD (de la Cuesta & Imai, 2016; Gelman & Imbens, 2019). Our main results thus rely on first-order polynomial local linear regression, and in several robustness checks we present the local linear and models with higher-ordered polynomials on either side of the threshold.

8
https://www.interieur.gouv.fr/Elections/Les-resultats/Municipales/

9 France is ranked 23rd (least corrupt) of 180 countries in TI CPI.

10
https://www.transparency.org/news/pressrelease/a_world_built_on_bribes_corruption_in_construction_bankrupts_countries_and

11
https://ec.europa.eu/regional_policy/sources/policy/how/improving-investment/public-procurement/study/country_profile/fr.pdf

12 More information on the red flags can be found in the Supplemental Appendix 1.

13 See Gasne et al. (2019) for local procurement law in France:
https://uk.practicallaw.thomsonreuters.com/8-502-1461?transitionType=Default&contextData=(sc.Default)

14 As municipalities vary in size and demand, not all municipalities have procurement contracts in each year. Thus, to maximize observations, we take the corruption risks aggregated over the whole mandate period.

15 For 2008 cases, the president was a center-right politician (Sarkozy) whereas, in 2014, the president was a center-left, (Hollande, Parti Socialist, PS).

16 We would lie to thank an anonymous reviewer at CPS for raising this point.

17 Our dependent variable in this case is: $Difference\;\;in\;\;corruption\;\;risk_{i}=corruption$. The distributions of the four groups are 36.5%, 46.5%, 12.7%, and 4.3%, respectively.

18 We elect to split the sample rather than create an interaction, as the results of Calonico et al. (2019) suggest that testing heterogeneous effects via interactions with the treatment variable produce inconsistent results.
