# Supplementary material for: Will Women Executives Reduce Corruption? Marginalization and Network Inclusion
Source: Comp Polit Stud. 2020 Dec 2;54(7):1292–322. doi: 10.1177/0010414020970218 (PMC8107401; doi:10.1177/0010414020970218)
Supplement: sj-pdf-1-cps-10.1177_0010414020970218 – Supplemental material for Will Women Executives Reduce Corruption? Marginalization and Network Inclusion [file sj-pdf-1-cps-10.1177_0010414020970218.pdf]

Will women executives reduce corruption? Marginalization and network inclusion

Appendix Section 1: The Dependent Variable: Corruption risks, red flags in procurement

The corruption risk data contain information on individual public procurement tenders, that is regulated administrative procedures in which public bodies purchase goods and services, for EU28 between 2005 and 2016, including for example contract value, the deadline for submitting bids and the assessment criteria used. They derive from the European Union's Tenders Electronic Daily (http://ted.europa.eu/), which is the mandatory online publication that falls under the remit of the Public Procurement Directive that aims to foster a Single Market in government contracts. This means that large, both national and EU, contracts are typically included in the database, with publication thresholds varying over time while being approximately 125 000 Euro for service contracts and 4 000 000 Euro for public works contracts. With respect to France, the database contains about 1.08 million contracts awarded for the entire period; however, we limit these to the cases where municipalities had a male/female top two candidate election. We also exclude any contract that was applied for at the national or EU level in order to focus on the procurement practices of the local level, our unit of analysis.

The data are structured by contract year. For our purposes, we construct municipal level procurement variables a by aggregating contracts awarded by local public bodies and agencies over the mayor's mandate period, using information on the location of the contracting entity (the municipality). Corruption risk measurement builds solely on those characteristics of the tendering processes that are in the hands of the local contracting entities within the boundaries of national and regional procurement laws, for example deciding on the deadline for submitting bids. While investments in a particular region by bodies located in another region may also influence the corruption level of that region, we focus only on the local decisionmaking powers when we define regional corruption levels.

We thus capture the high level corruption risk at the municipal level. Our measures tap into a deliberate restriction of open competition for government contracts in order to benefit a well-connected company, and we operationalize our dependent variable in two ways, differing only in the number of components included. As the majority of these indicators are more a representation of transparency than corruption (Baur et al. 2019), we limit the uses of these indicators to single bidding to best capture elite collusion and corruption. A contract that was awarded with one bidder is coded as a '1', and '0' if more than one competitor placed a bid.

Contracts are then aggregated to the municipality year (and municipality mandate period), and thus the dependent variable measures the proportion of single bid contracts awarded per mandate period in each observed municipality.

Appendix section 2: further description of independent and control variables

Figure A1: Sample Wide Single Bidding and Gender Means

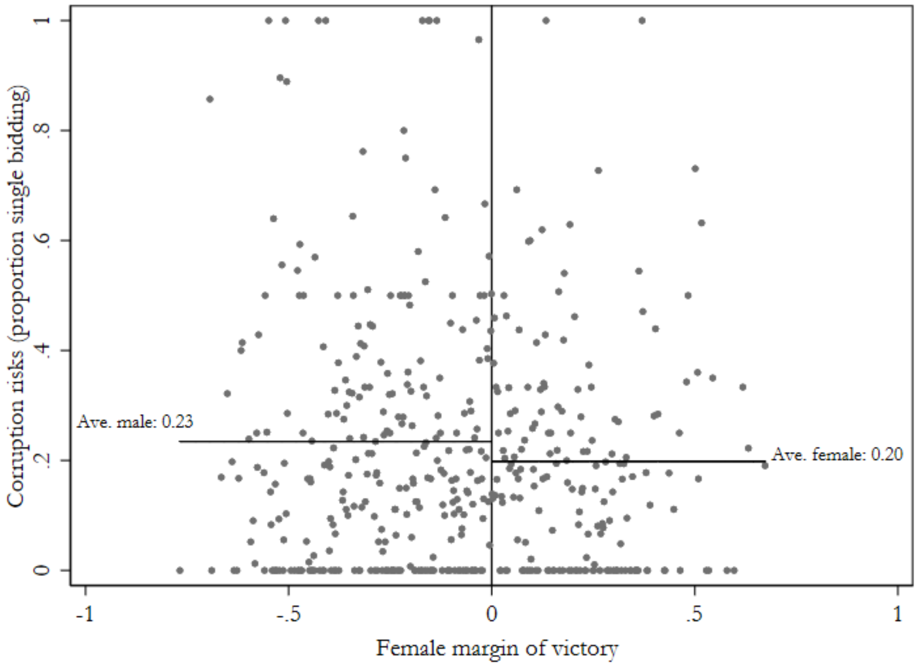

Table A1: Means and groups differences of covariates

Covariate	whole sample (1)	male mayors (2)	female mayors (3)	Difference: female-male, (p-value) (4)
% higher Education	26.3	26.7	25.5	0.08
	(10.9)	(10.9)	(10.4)	
Income per capita	2097.0	2120.3	2058.2	0.15
	(699.4)	(755.2)	(593.8)	
Income inequality	2.34	2.35	2.32	0.02
	(0.24)	(0.22)	(0.28)	
Unemployment rate	12.7	12.5	13.1	0.10
	(5.4)	(5.1)	(5.8)	
No. Firms (total)	831.2	813.3	861.6	0.63
	(1611.9)	(1449.7)	(1853.4)	
No. Firms (commercial)	93.2	92.2	94.7	0.82
	(182.1)	(164.4)	(208.6)	
Population	16990.3	17600.6	15959.1	0.38
	(31055.9)	(31832.1)	(29738.3)	
Population Density	1731.8	1853.1	1530.0	0.17
	(3788.9)	(4180.7)	(3020.9)	
Turnout. (r1)	61.8	61.6	62.1	0.25
	(7.8)	(7.8)	(7.9)	
Turnout (r2)	63.3	62.4	64.2	0.04
	(8.8)	(8.9)	(8.6)	
No. Party Lists	3.2	3.1	3.3	0.04
	(1.5)	(1.4)	(1.5)	
Age of Mayor	55.6	56.4	54.3	0.02
	(8.7)	(8.6)	(8.6)	
Incumbent	0.52	0.61	0.36	0.000
	(0.50)	(0.49)	(0.48)	
Mayor & President party aligned	0.76	0.76	0.77	0.77
	(0.43)	(0.43)	(0.42)	
% females on council	50.0	50.1	49.9	0.36
	(3.9)	(4.3)	(3.0)	
no. of Procurement contracts	11.6	11.7	11.4	0.92
	(45.1)	(50.0)	(35.1)	
Lagged corruption risks (t-1)	28.8	27.2	31.9	0.10
	(36.7)	(36.2)	(37.6)	

Note: means reported with standard deviations in parentheses. Difference of means significance test between female and male led municipalities reports a two-tailed p-value. Lagged corruption risks are the risks of corruption in the municipalities in the previous mandate period prior to the sitting mayor

i. *Municipality level data:*

In some cases, we were able to match the election year with data that temporally match the start year of a mandate period; in other cases, such data were not available, and thus we gathered the closest possible year, which in most cases was 2010.

unemployment rate –the unemployment rate (*taux de chômage*) in 2008 and 2014

Education – the % of residents with a tertiary degree (*Diplôme de l'enseignement supérieur)* or higher, 2010

Average salary – the average monthly wage in the municipality (*Salaire moyen* , Euros per month), 2010

Gender wage gap – the ratio of the average salary of a male / the average salary of the average female

Income inequality – the mean salary of the average executive (*Salaire moyen des cadres)* in a municipality / the average worker (*Salaire moyen des employés*), 2010

Population – the population of the municipality in 2008 and 2014

Population density – population/ kilometers^2

Health investments – i. number of doctors per 1000 inhabitants.
ii. number of nurses per 1000 inhabitants. 2010

Market competition:

Number of firms – number of total enterprises registered in the municipality in 2008 and 2014

Number of commercial firms – number of commercial firms (*Entreprises, Commercers*) registered in the municipality in 2008 and 2014

Number of service firms – number of service firms (*Services aux particuliers*) registered in the municipality in 2008 and 2014

Number of public firms - number of service firms (*Services publics*) registered in the municipality in 2008 and 2014

ii. Election variables[1]

vote share female: vote share (%) for the party led by the female candidate in the winning round

[1] All data come from official sources and match each election year: https://www.interieur.gouv.fr/Elections/Les-resultats/Municipales/elecresult__MN2014/(path)/MN2014/001/001007.html

vote share male: vote share (%) for the party led by the male candidate in the winning round

party of female – the political party led by the female candidate

party of male – the political party led by the male candidate

round 1 competition - number of total party lists in round 1

round 2 competition - number of total party lists in round 2

Winning round – whether the election was decided in round 1 or 2

Turnout – the electoral turnout in the round that decided the election (1 or 2)

Mayor's legislative power – total seats in the municipal council (*conseil municipal*) won by the mayor's party / the total number of council seats.

iii. *Mayor characteristics*

incumbency – was the mayor re-elected from the previous mandate period? (1=yes, 0=no)

newly elected – is the current mayor newly elected in their role as mayor? (1=yes, 0=no)

political experience – did the current mayor sit in the municipal council (legislature) in the previous mandate period?

Mayor age – age of the mayor (in years)

2nd place candidate age – the age of the runner up candidate heading the second largest list.

Mayor's previous occupation – the profession of the mayor prior to winning office

Appendix section 3: empirical test of design validity

The most common method of testing the 'as if random' assumption is to use parametric tests such as fourth-order polynomial regressions (Lee et al. 2008) or the difference of means tests for potential confounders within a small range around the threshold. In the close elections literature, the difference of group means is often within +/- 2 percentage points of the cut-off to highlight the similarities of the two groups right at the threshold (Lee et al. 2008; Butler 2009). Calonico et al. (2014) offer an improvement to this somewhat arbitrary choice of bandwidth, whereby they recommend the use of non-parametric, local linear regression with the use of data driven bandwidth selection where observations closer to the threshold are given more weight (Imbens and Kalyanaraman 2012; Calonico et al. 2014), which allows for direct testing of the continuity assumption. Testing the assumption regarding the (lack of) sorting at the cut-off is done via a density plot of the running variable to examine whether any discontinuities are observed at the threshold (Eggers et al. 2015).

Figure A2: Tests of Discontinuities among Covariates

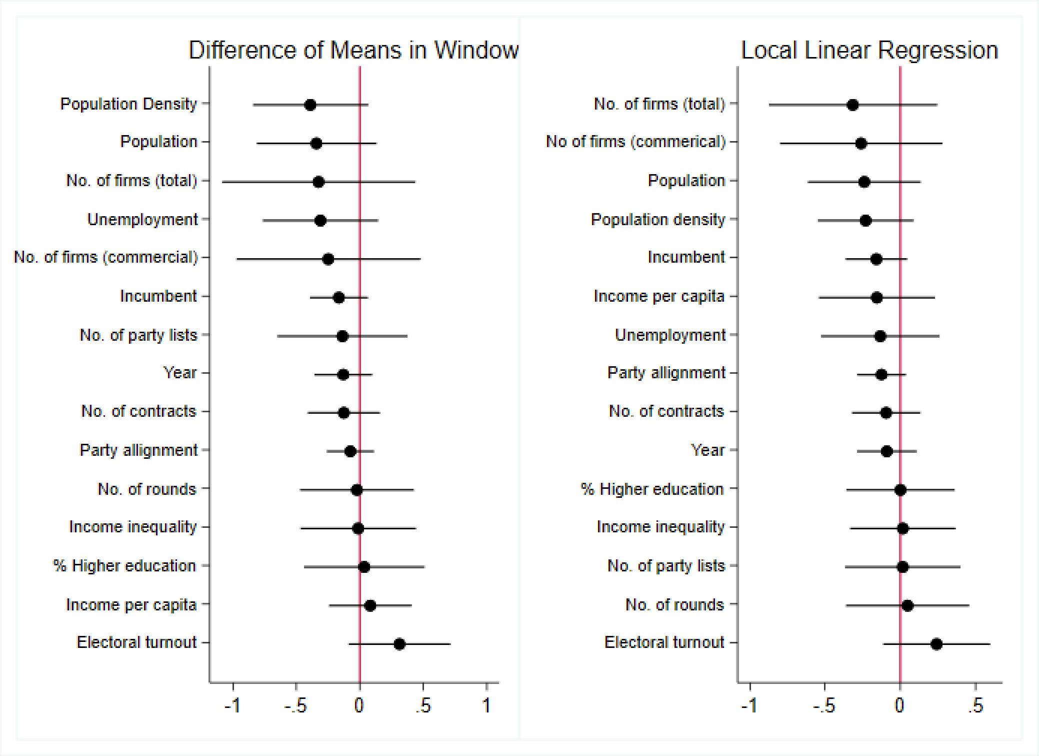

Note: tests of balance and discontinuities among covariates. For purposes of comparability, all non-binary variables have been standardized (z-scores). The first column is a difference of means test by within +/- 2% points of the cut-off margin of victory threshold (0). The second column is non-parametric local linear regression estimates of discontinuities at the threshold proposed by Calonico et al. (2014), using triangular kernels and mean square error (MSE) bandwidth selection from Imbens & Kalyanaraman (2012). 95% confidence intervals are shown around estimates.

More important than sample wide group means for the validity of the RD design are the characteristics of the two groups around the threshold (‘0’). Using the approach recommended by de la Cuesta and Imai (2016), in column one in Figure 2, we examine the mean differences in the male and female led towns among our covariates within a bandwidth of +/- 2% points in terms of margin of victory – that is to say, a test of the ‘as if random’ assumption. In the second column, we test for the continuity assumption with non-parametric local linear regression and flexible, data driven bandwidths for each estimate. For purposes of comparison, all non-binary covariates are standardized (z-scores), and estimates have a 95% confidence interval. In neither case do we find significant differences among our covariates, which suggests that the comparison of the groups at the threshold is valid according to the assumptions of the design.

Next, in Figure A3 we check for discontinuities of the sorting variable around the threshold. Overall, we observe a right-skewed distribution, as there is a significantly greater proportion of male mayors in the sample. However, around the ‘0’ mark, there does not appear to be any significant discontinuity on either side that would suggest electoral manipulation of any kind.

Figure A3: Histogram of Female Margin of Victory

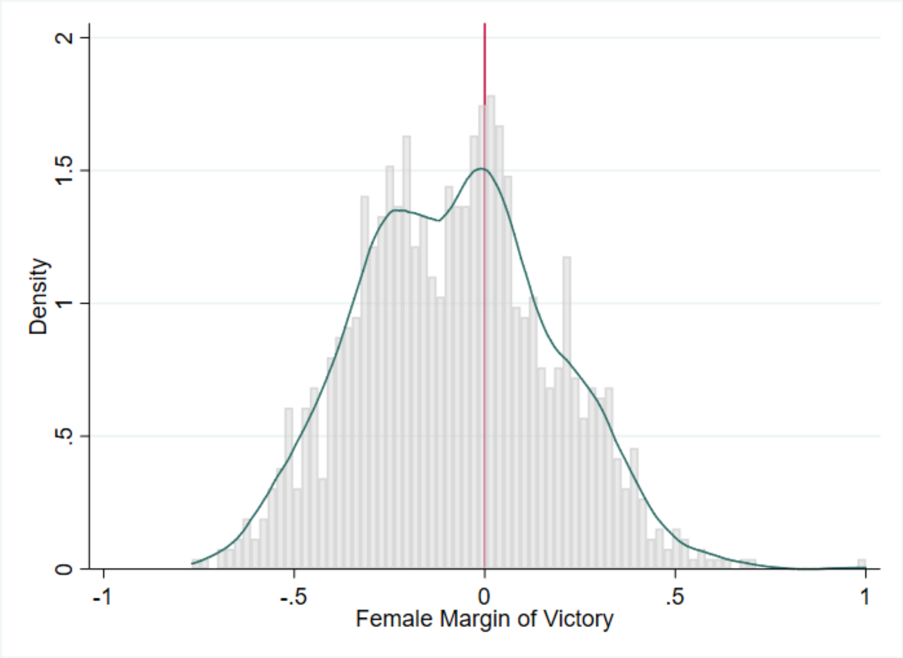

Finally, as recommended in the literature, we examine the means and discontinuities of the lagged dependent variable (Eggers et al. 2015). The lagged dependent variable in this case is an average of the corruption risk measure for the three years prior to the electoral victory of the

sitting mayor, weighted by the number of contracts per year (e.g. 2005-2007, and 2011-2013, for 2008 and 2014 election winners, respectively). Figure A4 examines the local linear effect lagged period and the current mandate period via a fitted RD plot in which the solid line is a fourth-order polynomial fit and the scattered dots are data driven, with optimally chosen binned means (Calonico et al. 2015). The local linear effects are estimated via coverage error rate (CER) bandwidth selection and show confidence intervals via robust estimation.

Figure A4: Female Margin of Victory and Lagged Corruption Risks: RD Plot

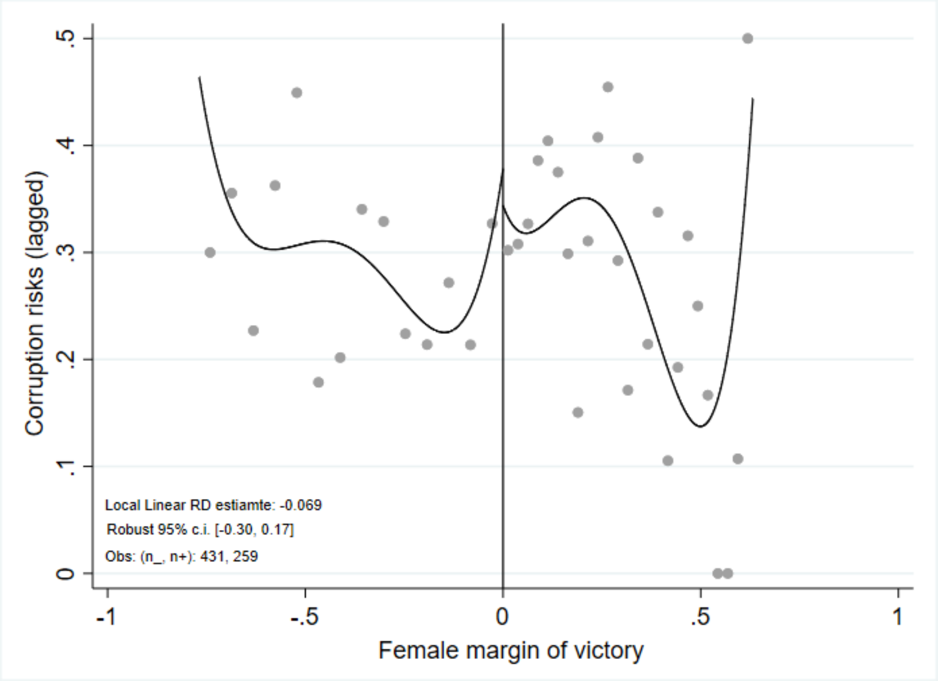

Note: Sample wide RD plot with equally spaced bins on either side of the threshold fit with a 4th polynomial order (quartic). The bandwidth is 1, which includes the whole sample. The x-axis shows the *female win* running variable with female mayors on the positive side and male on the negative side. The lagged risk for high level corruption is on the y-axis, measured as the proportion of contracts that are single bid per municipality-mandate period.

Figure A5: Tests of Discontinuities in the Lagged Dependent Variable – Full Sample and Sub-Groups

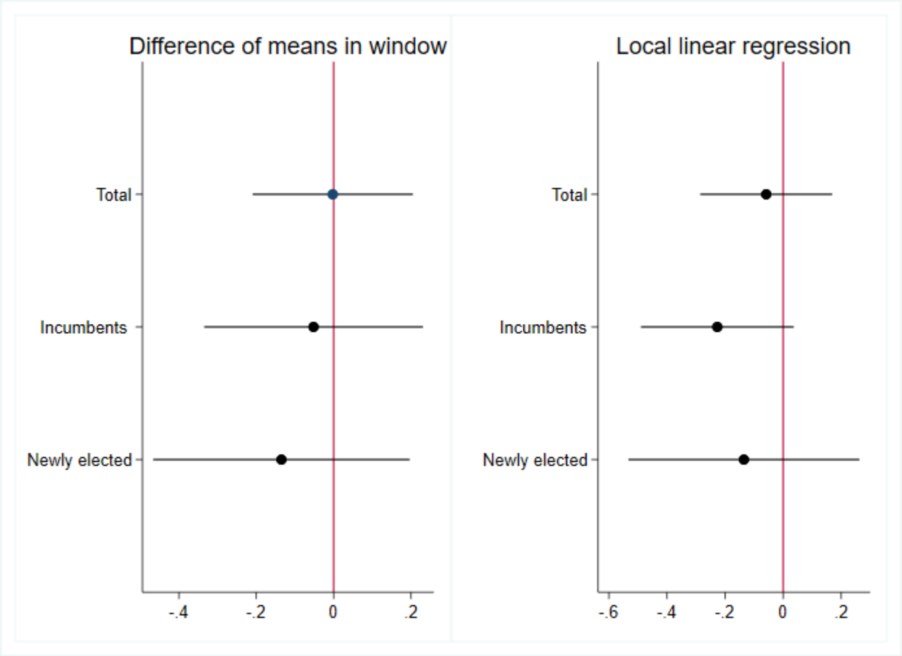

Note: tests of balance and discontinuities among the lagged dependent variable, which is an aggregate of the corruption risks for the three years prior to the 2008 and 2014 election, respectively. ‘incumbents’ and ‘newly-elected’ refer to the level of corruption risks in the sitting mayors’ municipality prior to the election analyzed. The first column is a difference of means test by within +/- 2% points of the cut-off margin of the victory threshold (0). The second column is non-parametric local linear regression estimates of discontinuities at the threshold proposed by Calonico et al. (2014), using triangular kernels and mean square error (MSE) bandwidth selection from Imbens and Kalyanaraman (2012). 95% confidence intervals are shown around estimates.

In Figure A5, we check the difference of means in a small window (plus or minus 3%) margin of victory on either side of the threshold) and the estimates for local linear regression for the whole sample as well as in the two sub-groups – incumbents and newly elected mayors. Although in all cases a negative effect of female victory is associated with a negative corruption risk score on average for the lagged measure of the dependent variable, we find in no cases that the effects are significant at the 95% level of confidence. The closest, however, is the local linear RD estimate in the incumbent group, suggesting that current female incumbents may have had fewer risks of corruption in their municipalities in their previous mandate period relative to incumbent male mayors.

Appendix section 4: RD Estimates Including Covariates

Building on Table 2 in the main text, we re-run the RD estimates to include the battery of covariates.

Table A2: Robustness Check of Table 2 in Main Text

Dep variable	Single Bidding					
Control function	None	Linear			Quadradic	Cubic
Bandwidth	All mixed-municipalities	h	h/2	2/h	h	h
	1	2	3	4	5	6
	Group 1: incumbent female mayors					
Female mayor	-0.046	-0.095	-0.157	-0.089	-0.145	-0.184
95% c.i. robust	[-0.131, 0.037]	[-0.269, 0.080]	[-0.483, 0.168]	[-0.261, 0.082]	[-0.419, 0.128]	[-0.528, 0.160]
95% ci. Bias-corrected		[-0.255, 0.066]	[-0.375, 0.060]	[-0.212, 0.033]	[-0.406, 0.114]	[-0.517, 0.149]
Bandwidth	1.00	0.167	0.083	0.334	0.127	0.147
Effective Obs.	648	217	124	526	176	198
Control Obs.	520	163	93	333	133	148
Treament Obs.	227	54	31	93	43	50
Mean single bidding	0.222	0.229	0.247	0.212	0.219	0.243
	Group 2: new female mayors					
Female mayor	-0.036	-0.159**	-0.25***	-0.148**	-0.191**	-0.202**
95% c.i. robust	[-0.081, 0.008]	[-0.309, -0.008]	[-0.459, -0.043]	[-0.300, 0.003]	[-0.361, -0.021]	[-0.381,-0.023]
95% ci. Bias-corrected		[-0.299, -0.019]	[-0.420, -0.083]	[-0.264, -0.032]	[-0.357, -0.024]	[-0.379,-0.025]
Bandwidth	1.00	0.105	0.053	0.210	0.118	0.168
Effective Obs.	701	218	115	358	228	295
Control Obs.	482	115	61	214	125	164
Treament Obs.	219	93	54	144	103	131
Mean single bidding	0.223	0.230	0.257	0.232	0.229	0.234
	Test of difference in coefficients between new females incumbent females					
T-test (p-value)	0.08	0.000	0.000	0.000	0.000	0.05

Note: column 1 displays results from simple OLS regression, while columns 2-4 are local linear regressions with first-order (linear) polynomials. Columns 5 and 6 use quadratic and cubic polynomials, respectively. The running variable is the margin of female mayoral victory with the sharp cut-off at '0'. Bandwidth ('h') is determined via coverage error rate (CER) with the aid of the data driven algorithm of Calanico et al. (2014), and all local linear regressions use triangular (e.g. weighted) kernel functions. Significance is determined by the 95% confidence interval of the estimate, with the first confidence interval clustered by municipality. The second confidence interval is the bias corrected estimate from Calanico et al. (2014). The number of observations is shown in total as are the number of observations on either side of the cut-off, with male mayors representing the control and female representing the treatment groups, respectively. The mean of the dependent variable in each model is presented in the final row. The t-test in the bottom panel summarizes a t-test of differences in coefficients across the two samples, testing whether the effect of new mayors ('n') is significantly different from incumbents ('i'), with the formula: $\beta_n - \beta_i / \sqrt{SE_n + SE_i}$. As H2 is directional, the p-value reported is one-tailed. ***p<0.01, **p<0.05, *p<0.10

We find results consistent with the previous split sample analysis from Table 2 in the main text – that gender effects with respect to corruption risks are driven by differences between newly elected women. While we find that the effect of incumbent women is indeed negative compared with male mayors, none of the RD estimates are significant. Conversely, each of the estimates comparing the effects of municipalities led by newly elected women versus all male led municipalities yields consistent effects. Newly elected female mayors have significantly lower corruption risks, and the treatment effect increases as the bandwidth decreases, demonstrating that the size of the difference is greater when analyzing the outcome among observations closest to the threshold. Moreover, we find that the differences in the estimates are significant across all RD models when control variables are included, demonstrating support for H2. In Table A3, we impose several bandwidths and polynomials to test the robustness of the results. The effect is significant for newly elected women in all models when considering bias corrected standard errors, and at or near 95% level significance for robust, clustered standard errors.

Figure A6: Separate RD plots of newly elected and incumbent mayors

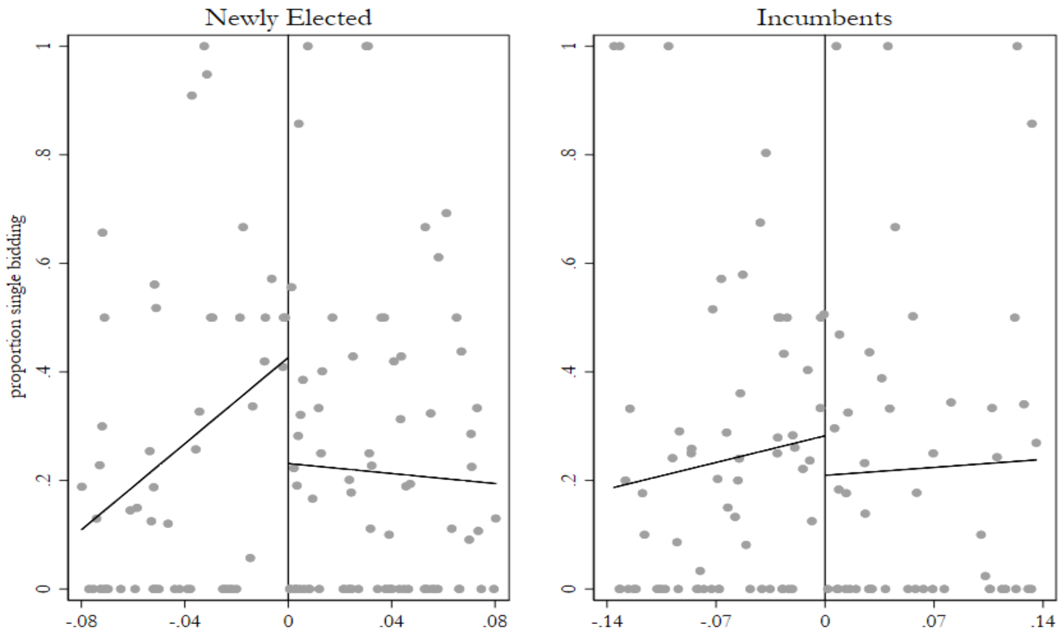

Note: RD plot with equally spaced bins on either side of the threshold fit with a first-polynomial order (linear) from column 2 in Table 2. The x-axis shows the *female win* running variable with female mayors on the positive side and the male on the negative side with all observations within the recommended bandwidths (see Table 2 for more details). The risk for high-level corruption is on the y-axis, measured as the proportion of contracts that are single bid per municipality-mandate period.

Table A3: Alternative Specifications

Bandwidth		0.25	0.1	0.05	CER
			I. All observations		W/ controls
Order of Polynomial					
	Linear	-0.054	-0.143**	-0.166**	-0.081
		(0.047)	(0.066)	(0.083)	(0.093)
	2nd	-0.126*	-0.182**	-0.23**	-0.165**
		(0.065)	(0.086)	(0.095)	(0.078)
	3rd	-0.181**	-0.201**	-0.368***	-0.186**
		(0.077)	(0.099)	(0.112)	(0.091)
	4th	-0.193***	-0.251**	-0.371***	-0.191**
		(0.064)	(0.104)	(0.143)	(0.095)
Observations	n-, n+	287, 252	125, 131	61, 81	
			II. Incumbents		
Order of Polynomial					
	Linear	-0.046	-0.123	-0.174	-0.081
		(0.078)	(0.124)	(0.174)	(0.093)
	2nd	-0.092	-0.181	-0.127	-0.128
		(0.112)	(0.194)	(0.271)	(0.138)
	3rd	-0.159	-0.151	-0.385	-0.204
		(0.149)	(0.255)	(0.255)	(0.184)
	4th	-0.197	-0.114	-0.577**	-0.254
		(0.187)	(0.383)	(0.215)	(0.211)
Observations	n-, n+	132, 77	51, 33	27, 24	
			III. Newly elected		
Order of Polynomial					
	Linear	-0.075	-0.201**	-0.218**	-0.176**
		(0.063)	(0.081)	(0.084)	(0.085)
	2nd	-0.183**	-0.253***	-0.315***	-0.221**
		(0.083)	(0.085)	(0.099)	(0.090)
	3rd	-0.248***	-0.251***	-0.444***	-0.239***
		(0.089)	(0.102)	(0.136)	(0.093)
	4th	-0.266***	-0.323***	-0.300*	-0.219**
		(0.091)	(0.112)	(0.157)	(0.098)
Observations	n-, n+	139, 165	71, 94	31, 57	

Note: results of local linear regression shown with standard errors clustered on municipalities in parentheses. In the fourth column, the bandwidth is determined via the CER method and control variables are included. The controls are population density, number of total registered firms, number of total registered commercial firms, year of election, incumbency, turnout, number of party lists, number of election rounds, % with higher education, monthly income per capita and unemployment rates. 'n-' and 'n+' are control and treatment observations, respectively. ***p<0.01, **p<0.05, *p<0.10, two-tailed test

Appendix section 5: First Difference Results, Full Tables

Table A4: Changes in Corruption Risks among newly elected mayors - Full results from Figure 4.

	(1)<br>Baseline model	(2)<br>Add control variables
Ref: male to male		
1.male to female	-0.025***	-0.025***
	(0.007)	(0.007)
2.female to male	0.003	0.004
	(0.008)	(0.010)
3.female to female	0.003	0.003
	(0.014)	(0.016)
Population density		0.000**
		(0.000)
Year		-0.003
		(0.007)
Turnout (in round won)		-0.000
		(0.001)
Lists (round 1)		0.003
		(0.003)
No of rounds		-0.019**
		(0.009)
Ave. higher Ed.		-0.000
		(0.001)
Wage inequality		0.008
		(0.017)
average wage		0.000
		(0.000)
No. of firms		-0.000
		(0.000)
No. of commercial firms		0.000
		(0.000)
constant	-0.077***	-0.072
	(0.004)	(0.051)
Obs.	542	483
R-squared	0.032	0.060

Standard errors are in parentheses, clustered by municipality
*** p<0.01, ** p<0.05, * p<0.1

Table A5: Effect of women on corruption risks among newly elected in 2008 in 1st versus 2nd mandate period - RD estimates from Figure 5

Type of RD estimation	(1)<br>1st mandate period	(2)<br>2nd mandate period
Conventional	-0.310*	0.134
	(0.170)	(0.121)
Bias corrected	-0.340**	0.117
	(0.170)	(0.121)
Robust	-0.340*	0.117
	(0.196)	(0.135)
n_, n+	19, 34	11, 27
h	0.092	0.126

Standard errors are in parentheses
*** p<0.01, ** p<0.05, * p<0.1

Appendix section 6: Further information about the French mayoral system

Mayors in France have executive authority over civil registration, culture, economy, education, environment, public order, roads, social welfare and urban planning, and is constant across all municipalities irrespective of population[2]. While they work in tandem with the council, the mayor and her deputies have responsibility for proposing a budget, creating and removing local public sector jobs, proposing municipal acquisition, grants and tax rates; which then rely on the voting majority from the council[3].

Municipal elections in France normally take place every six years for approximately 36,000 localities. The electoral system of local elections has taken on several reforms in recent years and varies depending on the municipality's population. As of the 2014 election, all localities with over 1000 inhabitants[4] employ a closed list proportional representation (PR) system with two rounds if necessary. In the case that a party wins an absolute majority, the election is limited to a single round; otherwise, all party lists that obtained greater than 10% of the vote continue to the second round. The plurality winner of the second round then wins majority control of the municipality council (*conseil municipal*), the size of which varies by the municipal population. The position of local mayor (*maire*) is then decided by the council and, with very few exceptions, awarded to the head candidate of the winning list[5]. Council seats are allocated via a method of majority voting with a proportional rate – thus even in a close election the seats are heavily skewed toward the winning list and there are thus no coalition governments at the local level, giving all mayors a strong political mandate. The mayor and council members

[2] The exception being Paris, in which the local police are administered by the central state.
[3] See the following for more details on the constitutional duties: https://www.legifrance.gouv.fr/affichCodeArticle.do?cidTexte=LEGITEXT000006070633&idArticle=LEGIARTI000020629575&dateTexte=20141108

have a fixed term of six years, and the mayor cannot be dismissed from office (Pawlowska and Radzik 2007).
